# Simple statistical models predict C-to-U edited sites in plant mitochondrial RNA

**DOI:** 10.1186/1471-2105-5-132

**Published:** 2004-09-16

**Authors:** Michael P Cummings, Daniel S Myers

**Affiliations:** 1 Center for Bioinformatics and Computational Biology, University of Maryland, College Park, MD 20742-3360, USA

## Abstract

**Background:**

RNA editing is the process whereby an RNA sequence is modified from the sequence of the corresponding DNA template. In the mitochondria of land plants, some cytidines are converted to uridines before translation. Despite substantial study, the molecular biological mechanism by which C-to-U RNA editing proceeds remains relatively obscure, although several experimental studies have implicated a role for *cis*-recognition. A highly non-random distribution of nucleotides is observed in the immediate vicinity of edited sites (within 20 nucleotides 5' and 3'), but no precise consensus motif has been identified.

**Results:**

Data for analysis were derived from the the complete mitochondrial genomes of *Arabidopsis thaliana*, *Brassica napus*, and *Oryza sativa*; additionally, a combined data set of observations across all three genomes was generated. We selected datasets based on the 20 nucleotides 5' and the 20 nucleotides 3' of edited sites and an equivalently sized and appropriately constructed null-set of non-edited sites. We used tree-based statistical methods and random forests to generate models of C-to-U RNA editing based on the nucleotides surrounding the edited/non-edited sites and on the estimated folding energies of those regions. Tree-based statistical methods based on primary sequence data surrounding edited/non-edited sites and estimates of free energy of folding yield models with optimistic re-substitution-based estimates of ~0.71 accuracy, ~0.64 sensitivity, and ~0.88 specificity. Random forest analysis yielded better models and more exact performance estimates with ~0.74 accuracy, ~0.72 sensitivity, and ~0.81 specificity for the combined observations.

**Conclusions:**

Simple models do moderately well in predicting which cytidines will be edited to uridines, and provide the first quantitative predictive models for RNA edited sites in plant mitochondria. Our analysis shows that the identity of the nucleotide -1 to the edited C and the estimated free energy of folding for a 41 nt region surrounding the edited C are the most important variables that distinguish most edited from non-edited sites. However, the results suggest that primary sequence data and simple free energy of folding calculations alone are insufficient to make highly accurate predictions.

## Background

RNA editing is the process whereby an RNA sequence is modified from the sequence corresponding to the DNA template. A particular form of RNA editing in plant mitochondria, by which some cytidines are converted to uridines before translation, occurs in many land plant lineages. Although cytidine to uridine conversion is most common, the reverse conversion is sometimes observed [1-4]. In plants, the phenomenon is best studied, albeit still poorly understood, in the mitochondria and plastids of angiosperms [5-8].

The majority of plant mitochondrial RNA editing occurs in coding sequences, and editing frequently changes codons, resulting in changes of amino acids, or, in some cases, creation of entirely new open reading frames [1,9,10]. These changes often result in an increase in similarity with respect to homologous protein sequences among different organisms (such as in wheat [11]), and Gray has postulated that the RNA editing process functions as a repair mechanism to correct otherwise-deleterious genomic mutations [12]. RNA editing has also been detected in introns, where it is conjectured to improve splicing efficiency [13].

The precise biochemical basis for C-to-U editing in plant mitochondria is unknown, although experimental evidence suggests a deamination reaction [14-18]. Despite substantial study, the molecular biological mechanism by which C-to-U RNA editing proceeds remains relatively obscure, although several experimental studies have implicated a role for *cis*-recognition [19-21]. The mechanism by which edited sites are recognized is also still poorly understood, but the importance of surrounding nucleotides has been noted [22]. A highly non-random distribution of nucleotides in the immediate vicinity of edited sites (within 10–20 nucleotides 5' and 3') is observed, but no precise consensus motif has been identified [9,16]. Additionally, previous studies suggest that inferred secondary structure is not important in site recognition for C-to-U conversion [16,19].

Identifying edited sites thus remains an open problem, one to which we have applied tree-based statistical models and an extension of such models. When applied to a similar problem (predicting peptide binding to major histocompatibility complex (MHC) class I molecules [23]), tree-based statistical methods generated very accurate models, identifying specific important residues when no precise sequence motif had previously been identified. Therefore, we were motivated to apply tree-based statistical models and an extension, random forests, to the problem of C-to-U RNA editing in angiosperm mitochondria using complete mitochondrial genome data for three species: *Arabidopsis thaliana*, *Brassica napus *and *Oryza sativa*. The objective for the current research was to identify sequence features that may provide insights into C-to-U editing of plant mitochondrial RNA. We address the following specific questions. Is there evidence that sufficient information exists within sequence regions flanking edited sites to accurately predict editing? Is there an association between estimated free energy of folding for short sequence regions containing edited sites and C-to-U editing? We report tree-based statistical analysis of three complete mitochondrial genomes and show that relatively simple models provide moderately accurate prediction of C-to-U edited sites.

## Results

### Tree-based statistical models

Analysis of each of the three species-specific mitochondrial genome data sets yielded substantially similar results (Table 1). Using flanking nucleotides and estimates of folding energy as predictor variables, the optimistic re-substitution-based estimates for cross-validated pruned models had a mean correct classification rate of 0.705 (sensitivity [the proportion of observations correctly identified as edited] 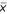 = 0.640, and specificity [the proportion of observations correctly identified as non-edited] 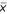 = 0.883) across the three species.

**Figure 1:**
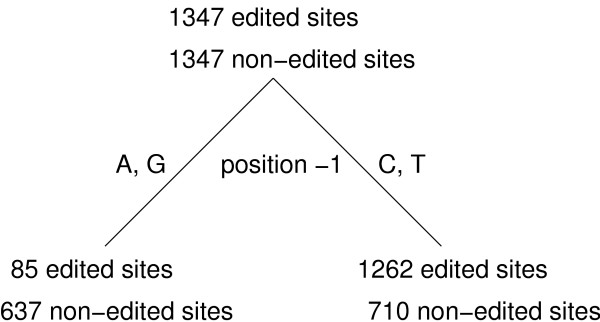
**Cross-validated pruned classification tree for the combined dataset. **The number of edited and non-edited sites are given at each node. The single split is based on the nucleotides at position -1 relative to the edited site.

As an additional classification tree analysis, we examined a dataset generated by combining the data from the three species. These results were generally similar to those described above for the mean of the individual genome datasets. The classification tree model is shown in Figure 1; the partition is defined based on the nucleotide immediately 5' (-1 position) of the edited/non-edited site. Of the 1972 observations with pyrimidine at the -1 position, 1262 (0.64) are edited and 710 (0.36) are non-edited sites. Of the 722 observations with purine at the -1 position, 85 (0.12) are edited and 637 (0.88) are non-edited sites.

### Random forests

**Figure 2:**
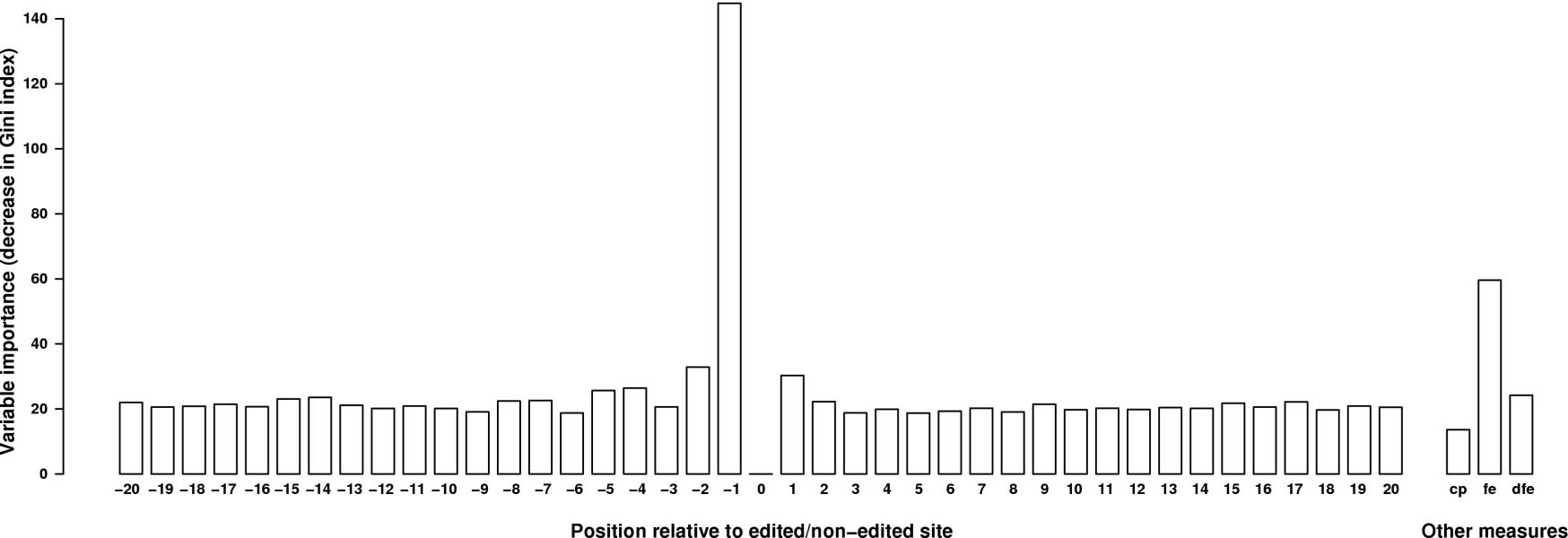
**Variable importance measures for the combined dataset. ** Numbered positions represent nucleotide state variables (with position zero representing the edited/non-edited site). The importance of each position is the decrease in the Gini index (a measure of impurity) induced by splitting the data on that position averaged over all trees (higher values are more important). The three variables based on estimates of free energy of folding are the codon position of the edited site (cp), estimated free energy of folding for the entire 41-nucleotide sequence centered on the edited/non-edited site (fe), and the difference in estimated free energy of folding between the edited and non-edited versions of the 41-nucleotide sequence (dfe).

Results from random forests (Table 2) were very similar to those obtained with classification trees and were somewhat more accurate. In single-species analyses, the mean accuracy rate was 0.744 (sensitivity 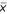 = 0.717, specificity 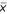 = 0.809). Analysis of the larger, combined data set yielded a model better than any of the single genome models with an accuracy of 0.848 (Table 2). Analysis of variable importance showed that the -1 position is overwhelmingly the most important factor in determining editing status. Other variables of lesser predictive value include estimated free energy of folding, and the -2 and +1 positions relative to the edited/non-edited site (Figure 2).

## Discussion

Despite their simplicity, the tree-based statistical models derived here performed moderately well, with mean accuracies across species generally ~0.71. Single trees were improved upon by constructing models based on ensembles of tree-based models (random forests) each of which was built using random subsamples of the data. This sub-sampling has the effect of reducing the variance through averaging and also reducing the correlation among models.

One of the advantages that random forests have over single classification trees is that they provide quantitative measures of variable importance, whereas with a simple classification tree, one is primarily limited to inferring variable importance from the frequency and location of the occurrence of variables in the model. One measure of variable importance is the decrease in the Gini index (a measure of impurity of observations at a particular node) induced by splitting on the variable, averaged over all trees [24].

In order to infer the relative importance of the predictor variables, we considered the measure of variable importance produced during the random forest run on the combined dataset, which is the most broadly representative dataset considered here. A plot of the variable importance measure for this dataset is shown in Figure 2; more important variables are shown as higher bars. The measure strongly indicates that the residue immediately 5' of the edited site (-1 position) is very important. These variable importance results are in agreement with previous work on C-to-U editing in mitochondria of *Arabidopsis thaliana, *which noted the -1, and -2 positions had highly non-random nucleotide distributions [9]. However, the results here differ from the past study of *Arabidopsis *in that we find no indication that the -17 position has much importance in edited site recognition. Also previously noted was that for 93.1% of the time [9], the -1 position contained a pyrimidine, which is the data partition found by the classification trees.

The free energy results contrast with previous studies indicating that secondary structure was not important in edited site recognition [16,19]. Our results show free energy is a relatively important variable in the random forest analyses. These results therefore indicate that secondary structure, as measured by free energy of folding for the 41 nt region centered on an edited/non-edited site, does help in distinguishing edited from non-edited sites. Previous studies determined putative secondary structures for mRNA regions containing edited sites and looked for conserved structural motifs. In contrast, we used estimates of free energy of folding, which are much easier to compare quantitatively. It may be that secondary or tertiary structure is even more important in determining edited sites than shown here; however, secondary structure may not be effectively represented by the calculated estimates of free energy of folding analyzed.

## Conclusions

Simple models based on nucleotides surrounding edited/non-edited sites and on estimated folding energies of those regions provide moderately accurate prediction of C-to-U RNA edited sites. More nuanced representation of secondary or higher-order structure in combination with variables based on the nucleotide positions found important here might improve models. Overall, the results strongly suggest that the C-to-U editing mechanism in plant mitochondria does not depend exclusively on the primary sequence immediately in the vicinity of the edited site.

## Methods

### Data sources

We obtained complete mitochondrial genome sequences and information regarding edited sites from GenBank [25] for three species: *Arabidopsis thaliana *(L.) Heynh. (mouse-ear cress), 455 edited sites, GenBank accession number NC_001284 [9]; *Brassica napus *L. (rapeseed), 425 edited sites, GenBank accession number AP006444 [26]; and *Oryza sativa *L (rice), 486 edited sites, GenBank accession numbers AB076665 and AB076666 [27]. None of the GenBank entries noted U-to-C RNA edited sites.

### Variable selection

Incomplete annotations in the GenBank sequences required us to algorithmically determine on which strand an edited site fell (the GenBank files sometimes supplied only a position number, with no strand information). The algorithm, implemented in a Perl script, scanned the entire GenBank file and built an in-memory representation of the layout of all genes and coding sequence regions in the genome. The strand with which an edited site was associated could then be determined by consulting the resultant genome map and checking which strand at the edited site contained a gene region. In no case were genes on both strands at an edited site, so strand localization was always unambiguous. In a few cases, however, a gene containing an edited site could not be located, or a site marked as a C-to-U edit did not contain a C in either strand. In these cases, the supposed edited site was eliminated from further consideration. Final numbers of included sites were as follows: *Arabidopsis*, 444; *Brassica*, 422; *Oryza*, 481. In total, 19 edited sites in the GenBank files were not included across all three species.

We also constructed a set of null observations of cytidines that are not edited to uridines. In constructing a null-set, it is important to ensure that the observations are as alike as possible to the edited observations (differing only in the trait to be measured), or the resulting model may be fictive. Here, our null-set observations were non-edited cytidines chosen at random from within gene regions of the genome. Additionally, we chose cytidines such that the null set had exactly the same distribution of codon positions as did the edited set, because the distribution of edited sites within the three possible positions of a codon is highly non-random with a bias to the first two positions [9] (Table 3).

For each observation, we recorded 40 nucleotide state variables: one variable for each of the 20 nucleotides sites 5' and 3' of the edited C (on the same strand). We chose a value of 20 for the number of nucleotides 5' and 3' so as to encompass the entire range of semi-conserved positions previously suggested, the most extreme of which occurs 17 bases 5' of the edited site [9]. In some cases other edited sites occurred within the 20 nucleotides 5' and 3' of the edited site used as a response variable. In these cases the edited sites as predictor variables were recorded as C. The low frequency of these sites at a particular position with respect to other edited sites results in non-significant effects, independent of how these sites are handled. In those cases where a full 20 nucleotides were not included within an annotated mRNA, the missing nucleotides were treated as unknown. Additionally, we included two variables based on free energy expressed in units of kcal/mole at 20°C: the estimated free energy of folding for each 41-nucleotide sequence (20 bases 5', the edited/non-edited base, 20 bases 3') and the change in free energy of folding between the non-edited and edited versions of the 41-nucleotide sequence. Free energies of folding were calculated using mfold [28,29] version 3.1 with program parameters except temperature at default values. Finally, we included codon position as a variable, even though the null set had been chosen so non-edited sites had the same distribution of codon position as the edited sites, as shown in Table 3. Including codon position as a predictor variable allows for possible interactions with other variables.

Finally, we created a combined data set to use alongside the species-specific datasets. The combined dataset is the result of combining all edited sites from all three species (there were no observations identical in all predictor variables), and then randomly selecting negative examples from the set of those already chosen for the three individual datasets. Negative examples were chosen to exactly match the positive examples in distribution over both species and codon position. The combined dataset comprises 2,694 observations.

### Data analysis

#### Tree-based statistical models

We used the R language for statistical computing [30], version 1.7.1 to conduct our analyses. Analyses included tree-based statistical models using rpart [31] and random forests using the FORTRAN implementation of random forest version 3.1 [24,32].

Tree-based statistical models [33], also known as classification and regression trees (CART) [34], are generated by recursively creating binary partitions of a dataset. Each partition is based on the value of a single predictor variable chosen to best produce homogeneous collections of a nominal or ordinal response variable (classification) or to best separate low and high values of a continuous response variable (regression). More precisely, the partitions may be considered as questions of the following form: Is the observation x_*i *_∈ *A*? Where *A *is a region of the variable space defined by some criterion of a single predictor variable. Answering such a question for all observations produces two groups: those observations for which the answer is *yes *(those in region *A*) and those for which the answer is *no *(x_*i *_∉ *A*, those in 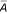). Subsequent binary partitioning continues until stopping criteria (variously defined) are met [34]. The result is a classification or a regression tree: a hierarchical series of data bifurcations that depicts the partition definitions and describes the resulting data subsets defined by each partition. To address concerns about possible over-fitting models to the data we used 10-fold cross-validation and pruned trees to the shortest within 1-*SE *of the best tree.

We assessed the significance of our tree-based statistical models through permutation where the predictor variables are randomized with respect to the response variable [35]. The frequency of observing a result value equal to or better than the observed value in 1 × 10^4 ^permutations is the estimate of the probability associated with the observed result.

#### Random forests

If one tree-based statistical model is good, then an ensemble (forest) of appropriately constructed tree models should be even better, which is the principal idea of random forests. A random forest attempts to improve upon a simple tree-based statistical model by generating a collection of such models and using them in aggregate [24,32]. Each model in a random forest is generated from a bootstrap sample of the original dataset, and at each node in each model a search for the best possible split is through a subset of variables selected at random from the bootstrap sample of predictor variables. These randomization steps decrease prediction error through variance reduction resulting from averaging and by decreasing the correlation between individual models in the ensemble [36,37]. Each of our random forest analyses comprised 1 × 10^4 ^individual models constructed by sub-sampling seven predictor variables at each node.

Several model summary statistics were calculated, including sensitivity, which is the proportion of observations correctly identified as edited, specificity, which is the proportion of observations correctly identified as non-edited, and accuracy, which is the total proportion of observations correctly identified. More formally, these definitions are:

*sensitivity = true positives/*(*true positives *+ *false negatives*);

*specificity = true negatives/*(*true negatives *+ *false positives*); and

*accuracy *= (*true positives *+ *true negatives*)/*total*.

## Authors' contributions

MPC conceived, designed and coordinated the study. DSM carried out the programming and statistical analyses. Both authors wrote and approved the final manuscript.

## Supplementary Material

Additional File 1 *Arabidopsis thaliana ***data file **File is plain text, space delimited. First row is column headings with variable names: edit; + site is edited, - site is not edited; -20 through 20, nucleotide position relative to edited site; cp, codon position; fe, estimated folding energy; dfe, difference in estimated folding energy between pre-edited and edited sequences; and loc, location of focus site (position 0) in GenBank file. Each subsequent line represents a observation. Click here for file

Additional File 2 *Brassica napus ***data file **File is plain text, space delimited. First row is column headings with variable names: edit; + site is edited, - site is not edited; -20 through 20, nucleotide position relative to edited site; cp, codon position; fe, estimated folding energy; dfe, difference in estimated folding energy between pre-edited and edited sequences; and loc, location of focus site (position 0) in GenBank file. Each subsequent line represents a observation. Click here for file

Additional File 3 *Oryza sativa ***data file **File is plain text, space delimited. First row is column headings with variable names: edit; + site is edited, - site is not edited; -20 through 20, nucleotide position relative to edited site; cp, codon position; fe, estimated folding energy; dfe, difference in estimated folding energy between pre-edited and edited sequences; and loc, location of focus site (position 0) in GenBank file. Each subsequent line represents a observation. Click here for file

Additional File 4 **Combined data file **File is plain text, space delimited. First row is column headings with variable names: edit; + site is edited, - site is not edited; -20 through 20, nucleotide position relative to edited site; cp, codon position; fe, estimated folding energy; dfe, difference in estimated folding energy between pre-edited and edited sequences; and loc, location of focus site (position 0) in GenBank file. Each subsequent line represents a observation. Click here for file

## Figures and Tables

**Table 1:** Summary statistics for tree-based statistical models.

	Accuracy	Sensitivity	Specificity
*Arabidopsis thaliana*	0.711	0.645	0.888
*Brassica napus*	0.693	0.630	0.887
*Oryza sativa*	0.709	0.645	0.874
combined	0.705	0.640	0.882

**Table 2:** Summary statistics for random forest models.

	Accuracy	Sensitivity	Specificity
*Arabidopsis thaliana*	0.744	0.701	0.811
*Brassica napus*	0.765	0.733	0.808
*Oryza sativa*	0.722	0.716	0.808
combined	0.848	0.823	0.877

**Table 3:** Counts of C-to-U edited sites for each codon position.

	Codon Position			
		
Species	1	2	3	Not in Codon
*Arabidopsis thaliana*	149	231	51	13
*Brassica napus*	142	243	33	4
*Oryza sativa*	174	230	77	0
